# Some American Hospitals

**Published:** 1892-02-06

**Authors:** 


### Some American Hospitals.

By Our Special Commissioner.

### VI.—THE PHILADELPHIA HOSPITAL, BLOCKLEY.

This hospital is the Poor Law Infirmary of the City of Philadelphia. It is under the control and management of the municipal authorities. It has a special interest for English people, because it was the scene of Miss Fisher's labours, who died at her post here in 1888, after doing a piece of good work which will be memorable for all time in the history of the medical institutions of the United States. The buildings are old and the wards have been constructed upon a plan commonly met with in Russia. It is the worst type of corridor pavilion, its chief feature being one long enclosed corridor into which each of the wards open. There is an inadequate, and indeed, improper provision for the lavatories and water-closets, which ought to be entirely re-constructed without a moment's delay. The wards, which are old, present no special features of interest except (thanks to the excellent supervision of the heads of the nursing department) that they are surprisingly clean and in first rate order. About eight thousand patients are treated at this hospital. The average daily population being about one thousand throughout the year. In connection with the hospital is an insane department for pauper cases, into which some nine hundred patients are crowded, it being frequently necessary to make up beds on the floor. We understand that the whole arrangements for the treatment of these lunatics is inadequate, and the Municipality has determined to erect new buildings, consisting of two ward blocks, bath rooms, wash rooms, &c., so we may hope that steps are being taken in the right direction to modify existing evils. The whole Institution is under the medical care of Dr. D. E. Hughes, chief resident physician, who rightly claims that the recent decision to place the hospital for the insane under the medical control and management of the chief resident physician of the hospital, instead of dividing the authority between the members of the visiting staff, is well calculated to produce satisfactory results. It would appear that in the insane department there are few recoveries, as 283 new cases were admitted during the past year, and only 227 were discharged, or died. Arrangements are being made to give lectures upon the care of the insane to nurses and attendants, which is a good feature. Mechanical restraint is used here, seclusion being also employed, but neither are resorted to, except by the direction of a medical officer. The night service is in charge of a competent night attendant with a night patrol, "who visit each ward every hour between 9 p.m. and 6 a.m., making a written record of what each patient is doing at the time visited, such records being filed in the department." The medical staff are greatly hampered in the treatment of their cases by the absence of adequate facilities for the employment of the patients, the average daily number of lunatics employed during the year having been only eighty males and a hundred females, although double that number would have been benefited had it been possible to set them to work in a useful manner.

### Nursing Department.

This department is in charge of two very able women, Miss Marion E. Smith, chief nurse, and Miss Roberta M. West, her assistant. Everything that devoted service and whole-hearted attention to duty can accomplish to make the Philadelphia hospital efficient is done by these two ladies. They are, however, crippled for want of necessary appliances and room. The class room in which the lectures are given to the nurses is, not only too small, but in every way inadequate for the purpose. The sleeping accommodation for the nursing staff is a serious reflection upon those who are responsible for the management of the hospital, and now that attention is called to the fact, a weight of responsibility will rest upon the whole of the residents in Philadelphia, if they longer permit the work of the nursing department to be handicapped and rendered largely inadequate, for the want of a suitable nursing home for the accommodation of the staff. The Pennsylvania hospital is said to possess the largest nursing school in the whole of the United States, and if this be true, it is monstrous that it should possess neither a nursing home, nor adequate buildings for the training and instruction of the many devoted women who resort to it, in order to become skilled and capable nurses. Politics and inefficiency seem to be synonymous terms in many places in the United States, and we gathered from much that we saw and heard that politics in the past has been the curse of the Philadelphia Hospital. But we hope, having regard to the fact that the citizens have permitted an enormous sum to be spent upon the municipal buildings of the town, that they will not rest until they have an assurance, that the City Council have voted the necessary means, and are making every possible haste to erect a suitable building for housing and instructing the nursing staff. It is surely a little incongruous to erect memorials to the late Miss Fisher, and to continually talk of her work, whilst those in authority remain indifferent to the needs of her successors, and by their inaction place the nurses, whom Miss Fisher loved so well, in a position which is largely dangerous to life and wholly opposed to justice and to a sense of what is due from employer to employed. One little point will show any visitor how inadequate is the accommodation. Let him inspect the class-room where the nurses are lectured, and then pay a visit to the diet kitchen and see the sort of appliances and the general arrangements therein for teaching the nurses sick diet cookery. Let him then run over to the Johns Hopkins Hospital, Baltimore, and see what are the minimum requirements which the experienced authorities of a modern hospital feel to be necessary to enable pupil nurses to be adequately taught and trained in nursing, cooking, and other matters. We do not think the sturdy Presbyterian fathers of Philadelphia could hold up their heads again if they permit politics, or indeed anything to impede the progress and efficiency of this great Hospital and Nurses' Training School. Prompt action is certainly called for, and we hope to see its results promptly.

Mr. George W. Childs and others have taken so much interest in the Philadelphia Hospital that we hope, now that attention has been called to the inadequate arrangements, that steps will be taken to apply a drastic remedy without delay. Meanwhile, we congratulate the muncipality upon the efficiency of the nursing staff and its chie s who are entitled to high praise for what they have already accomplished with very inadequate means.

